# Five-years’ prognostic analysis for coronary artery ectasia patients with coronary atherosclerosis: A retrospective cohort study

**DOI:** 10.3389/fcvm.2022.950291

**Published:** 2022-10-11

**Authors:** Ruifeng Liu, Xiangyu Gao, Siwen Liang, Huiqiang Zhao

**Affiliations:** Department of Cardiology, Beijing Friendship Hospital Affiliated to Capital Medical University, Beijing, China

**Keywords:** coronary artery ectasia (CAE), coronary heart disease (CHD), acute myocardial infarction (AMI), major cardiovascular events (MACE), prognosis

## Abstract

**Background:**

Most of coronary artery ectasia (CAE) patients have comorbid coronary atherosclerosis. It was lack of prognostic data for CAE patients with coronary heart disease (CHD) and for whom with acute myocardial infarction (AMI).

**Objective:**

To determine the overall prognosis for CAE patients.

**Materials and methods:**

This study was a retrospective cohort study. Fifty-one patients with CAE and comorbid AMI (CAE + AMI) and 108 patients with CAE and comorbid CHD (CAE + CHD) were enrolled and matched to non-CAE subjects at a ratio of 1:3 using a propensity score method, respectively. Controls for CAE + AMI group were 153 AMI patients, controls for CAE group were 324 CHD patients and 329 participants with relatively normal coronary arteries (CON). We followed them up to observe major cardiovascular events (MACE).

**Results:**

The Kaplan-Meier curves showed that the prognosis in CAE + AMI group was worse than in AMI group (5-year non-MACE rate: 62.70% vs. 79.70%, *P* = 0.010), the prognosis in CAE group was worse than in CHD and CON groups (5-year non-MACE rate: 74.10% vs. 85.80% and 96.70%, respectively, *P* = 0.000). The main MACEs in CAE + AMI and CAE groups were AMI reoccurrence (19.61% vs. 4.57%, *P* = 0.002) and re-hospitalization due to repeated angina pectoris (14.81% vs. 8.33% and 2.74%, *P* = 0.000), respectively. Additionally, the COX regression analysis revealed that the protective factors for preventing MACE in CAE + AMI group included antiplatelet agents (hazard ratio = 0.234, *P* = 0.016) and angiotensin-converting enzyme inhibitor/angiotensin receptor inhibitor (ACEI/ARB, hazard ratio = 0.317, *P* = 0.037). Whereas the main factor promoting MACE in CAE group was the degree of coronary stenosis (Gensini score, hazard ratio = 1.011, *P* = 0.022).

**Conclusion:**

The prognosis of patients with CAE + AMI was worse than that of those with AMI. The overall prognosis of patients with CAE was worse than that of those with CHD. CAE + AMI and CAE groups had different characteristics; the former was prone to AMI reoccurrence, and the latter was prone to repeated angina pectoris. To prevent MACE, medications, including antiplatelets and ACEI/ARBs, are indicated for patients with CAE + AMI, whereas prevention of the progression of atherosclerotic lesions is indicated for patients with CAE.

## Introduction

Coronary artery ectasia (CAE) refers to the dilatation of the coronary arteries to a diameter more than 1.5 times of its normal adjacent segment (1). Its prevalence is 1.2–7.4% among patients who have undergone coronary angiography (2). Comorbidity of coronary heart disease (CHD) occurred in more than 80% of patients with CAE (3). Its pathological manifestations are characterized by the extensive destruction of musculoelastic elements, particularly elastin fibers, which are dominant components of the extracellular matrix of the coronary wall (4, 5). It leads to slow blood flow and dysfunction in microcirculation (6, 7), thrombosis in the expanded coronary (3), and increased rupture risk of the expanded part (8). Therefore, the main clinical manifestations include angina, acute myocardial infarction (AMI), arrhythmia, and sudden death (2, 9, 10). In our clinical practice, some patients with CAE present with angina pectoris as the main manifestation, whereas others are more likely to present with AMI. Due to the low prevalence rate, it was still lack of the overall prognosis data for patients with CAE.

In this study, patients with coronary artery dilatation hospitalized in Beijing Friendship Hospital in recent years were followed up to observe their main cardiovascular events (MACE). These results would be useful for clinical practice and future research in this field.

## Population and method

### Patient population

**FIGURE 1:**
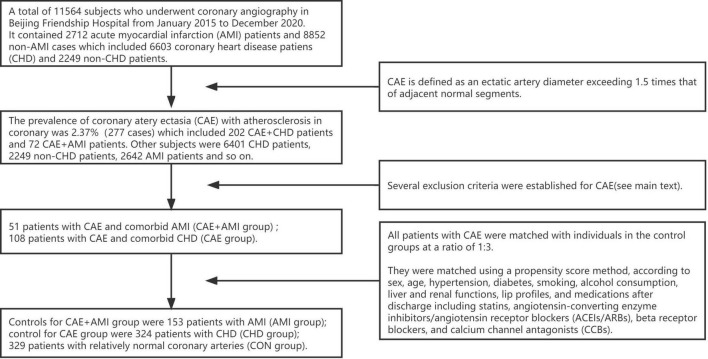
The flowchart for subjects enrollment.

This study was a retrospective cohort study, the process of subject enrollment was showed in Figure 1. Fifty-one patients with CAE and comorbid AMI (CAE + AMI group) and 108 patients with CAE and comorbid CHD (CAE group) were selected from the population of patients who underwent coronary angiography in Beijing Friendship Hospital from January 2015 to December 2020. All patients with CAE were matched with individuals in the control groups at a ratio of 1:3. They were matched using a propensity score method, according to sex, age, hypertension, diabetes, smoking, alcohol consumption, liver and renal functions, lip profiles, and medications after discharge including statins, angiotensin-converting enzyme inhibitors/angiotensin receptor blockers (ACEIs/ARBs), beta receptor blockers, and calcium channel antagonists (CCBs). Finally, the controls for the CAE + AMI group were 153 patients with AMI (AMI group); those for the CAE group were 324 patients with CHD (CHD group); and 329 patients with relatively normal coronary arteries (CON group). This study was approved by the ethics committee of Beijing Friendship Hospital (2017-P2-013-02) and conducted in accordance with the Declaration of Helsinki. All study participants provided written informed consent.

### Inclusion criteria

Coronary artery ectasia (CAE) is defined as an ectatic artery diameter exceeding 1.5 times that of adjacent normal segments (4). CHD is defined as stenosis of ≥50% in one or more major coronary arteries (11). Participants with stenosis of <20% in their coronary arteries were used as controls. According to current consensus (12), AMI is defined by an elevated cardiac biomarker and at least one of the following: (1) symptoms relating to ischemia; (2) changes on an electrocardiogram, such as ST segment changes, new left bundle branch block, or Q waves; and (3) changes in the motion of the heart wall on imaging. Since our preliminary analysis showed that CAE + AMI might be different from CAE (the formal data in this research confirmed it), patients with old myocardial infarction were not included in the CON, CHD, and CAE groups.

### Exclusion criteria

Several exclusion criteria were established. These were diagnosis of cardiomyopathy, valvular heart disease, heart failure, aneurysm in other vessels, collagen tissue diseases, vasculitis, syphilis, chronic obstructive lung disease, pulmonary hypertension, early menopause, documented history of hepatic diseases, renal failure, known malignancy, local or systemic infection, previous history of infection (<3 months), and other acute or chronic inflammatory diseases.

### Basic clinical characteristics

The hospital medical records were detailed and intact. Most of the baseline data in this research were extracted from the medical records; they included demographic data (age and sex), disease history (CHD, diabetes, and other diseases), history of smoking and alcohol consumption, family history of disease (hypertension, diabetes, and CHD), and medications taken before admission and after discharge. The body mass index (BMI) was calculated by dividing weight in kilograms by height in meters squared (kg/m^2^).

For patients with AMI (AMI and CAE + AMI groups), the myocardial infarct size could be estimated by peak blood concentrations of cardiac-specific enzymes, including creatine kinase MB fraction (CK-MB), myoglobin (Myo), and troponin I (TnI) (13). Left ventricular function was gauged by echocardiography, peak value of N-terminal pro-brain natriuretic peptide (NT-proBNP), and Killip grades.

### Biochemical indicators

The elbow vein blood was extracted during the next morning after admission and sent to the laboratory of Beijing Friendship Hospital to detect serum levels of glutamic pyruvic transaminase (ALT), serum creatinine, urea nitrogen, total cholesterol (TC), triglyceride (TG), high-density lipoprotein cholesterol (HDL-c), low-density lipoprotein cholesterol (LDL-c), and other serum components. For patients with AMI, serum concentrations of TnI, Myo, CK-MB, and NT-proBNP were measured at admission and at 12-h intervals during the first 5 days after presentation of AMI (from symptom onset).

### Echocardiography and coronary angiogram analysis

Transthoracic echocardiography was performed after in-hospital admission and at a median of 5 days after AMI. All images were analyzed by a single investigator, who was blinded to all clinical data. The coronary angiography was performed using a radial artery approach or a femoral artery approach, and each image was interpreted by two independent cardiologists.

Most patients with ST-segmental elevated myocardial infarction (STEMI) received an emergency percutaneous coronary intervention (PCI) as part of reperfusion therapy within 12 h of the onset of symptoms. For most patients with non-STEMI, initial antithrombotic therapy was instituted, and subsequent coronary angiography (delayed PCI) was performed within the first week.

According to the Markis classification method (4), CAE could be classified into four groups based on the extent of ectasia in the coronary arteries: Markis type I, diffuse ectasia of two or three vessels; Markis type II, diffuse disease in one vessel and localized disease in another vessel; Markis type III, diffuse ectasia of one vessel only; and Markis type IV, localized or segmental ectasia (4).

The Gensini scoring method (14) was used to evaluate the extent of coronary stenosis, and the most severe stenotic site was considered as the stenotic site for scoring. A stenotic diameter of <25% was scored as 1 point, 25–49% as 2 points, 50–74% as 4 points, 75–89% as 8 points, 90–99% as 16 points, and total occlusion as 32 points. The above scores were multiplied by a corresponding coefficient: 5 for the left main branch (LM); 2.5 and 1.5 for the proximal and middle segment of the left anterior descending artery (LAD), respectively; 1 and 0.5 for D1 and D2 in the diagonal branches, respectively; 2.5 and 1 for proximal and distal segment lesions of the left circumflex artery (LCX), respectively; and 1 for proximal, middle, distal, and posterior descending branch lesions of the right coronary artery (RCA). The sum of the scores for each lesion was the total score of the degree of coronary artery stenosis for a patient.

### Clinical endpoints and follow-up

Our research team followed up on participants at the outpatient department and with telephones at 1, 3, 6, 12, 24, 36, 48, and 60 months after discharge. Early or delayed follow-up was within a week of the scheduled time point. The missing data were addressed with propensity score methods. The endpoint was MACE; in this study, it referred to cerebral infarction, cerebral hemorrhage, cardiac death, major hemorrhagic events, malignant arrhythmias, cardiogenic shock, readmission, revascularization therapy, and AMI.

### Statistical analysis

The SPSS 26.0 statistical software (IBM Corp., Armonk, NY, USA) was used for all statistical analyses. Normally distributed continuous data were described using X ± SD, and their inter-group difference comparisons were performed using independent *T* test analysis. Discrete data were summarized as frequencies and analyzed using the χ2 test for inter-group differences. The multi-group comparisons were performed using the one-way ANOVA and rank-sum test. Non-normally distributed continuous data were summarized using the median and inter-quartile range. The 5-years’ prognostic data for all participants (AMI group, AMI + CAEgroup; CON group, CHDgroup, CAE group) was analyzed by the Kaplan-Meier curve; the COX regression analyses was used to find out factors relating to CAE (CON = 1, CHD = 2, CAE = 3) and CAE + AMI (AMI = 0, AMI + CAE = 1); the logistic regression was aimed to find out the independent factors relating to CAE + AMI, in this regression model, the dependent factor was CAE (CAE + CHD = 0, CAE + AMI = 1). *P* < 0.05 indicated statistically significant difference.

## Results

According to Table 1, the following baseline characteristics of all participants were observed. (1) Comparing the CAE + AMI and AMI groups, number of old cases of myocardial infarction was higher in the CAE + AMI group than in the AMI group. However, no significant differences were observed between both groups for most baseline data. (2) No significant differences in baseline characteristics were observed among the CAE, CHD, and CON groups. (3) The CAE + AMI group had higher number of males, lower hypertension rate, lower CHD rate, higher liver function and renal function, and poorer lipid profile than the CAE group.

**TABLE 1:** Baseline characteristics of participants.

	AMI (*n* = 153)	CAE + AMI (*n* = 51)	*P1*	CON (*n* = 3290	CHD (*n* = 324)	CAE (*n* = 108)	*P2*	*P3*
Age, years	62.00 (55.00–75.00)	63.00 (55.00–74.00)	0.948	63.00 (57.50–69.50)	65.00 (59.00–70.00)	65.00 (58.25–71.00)	0.944	0.461
Male, n (%)	119 (77.78%)	41 (80.39%)	0.948	122 (37.08%)	106 (32.72%)	36 (33.33%)	0.478	0.000
Hypertesion, n (%)	89 (58.17%)	30 (58.82%)	0.935	237 (72.04%)	246 (75.93%)	83 (76.85%)	0.427	0.019
SBP, mmHg	133.00 (120.00–147.00)	122.50 (116.00–140.50)	0.141	130.00 (120.00–140.00)	130.00 (120.00–140.00)	130.00 (120.00–140.00)	0.479	0.560
DBP, mmHg	74.00 (65.00–82.50)	73.00 (70.00–81.25)	0.634	79.00 (70.00–85.00)	79.00 (70.00–85.00)	76.00 (70.00–86.00)	0.439	0.398
Diabetes, n (%)	49 (32.03%)	16 (31.37%)	0.931	89 (27.05%)	110 (33.95%)	34 (31.48%)	0.157	0.570
HbA1c,%	5.90 (5.50–6.53)	6.20 (5.30–6.75)	0.969	5.90 (5.50–6.50)	6.00 (5.60–6.70)	6.10 (5.70–7.00)	0.269	0.509
Fasting glucose, mmol/L	5.68 (4.84–7.62)	6.46 (4.65–6.75)	0.562	5.20 (4.81–5.96)	5.38 (4.81–6.22)	5.44 (4.88–6.38)	0.751	0.234
Smoking, n (%)	85 (55.56%)	31 (60.78%)	0.514	158 (48.02%)	154 (47.53%)	48 (44.44%)	0.807	0.054
Alchohol, n (%)	59 (38.56%)	21 (41.18%)	0.741	133 (40.43%)	107 (33.02%)	35 (32.41%)	0.099	0.280
CHD, n (%)	49 (32.03%)	13 (25.49%)	0.785	–	206 (63.58%)	66 (61.11%)	0.645	0.010
OMI, n (%)	14 (9.15%)	12 (23.53%)	0.008	–	–	–	–*	–
CHD family history, n (%)	53 (34.64%)	9 (17.65%)	0.022	81 (24.62%)	88 (27.16%)	28 (25.93%)	0.760	0.249
Hypertension family history, n (%)	44 (28.76%)	8 (15.69%)	0.064	85 (25.84%)	81 (25.00%)	32 (29.63%)	0.634	0.059
Diabetes family history, n (%)	19 (12.42%)	7 (13.73%)	0.808	42 (12.77%)	39 (12.04%)	9 (8.33%)	0.459	0.291
BMI (kg/m^2^)	25.95 (23.38–29.01)	25.80 (23.00–28.41)	0.785	26.00 (25.00–29.00)	26.50 (25.00–29.00)	26.50 (25.00–29.00)	0.953	0.156
Heart rate,bpm	73.00 (64.00–83.00)	68.50 (61.75–79.25)	0.125	70.00 (63.00–79.00)	69.00 (62.00–76.00)	68.00 (61.00–73.75)	0.313	0.382
ALT, U/L	22.00 (15.00–33.00)	21.00 (15.00–38.00)	0.809	18.00 (14.00–24.00)	17.00 (13.00–24.00)	17.00 (13.00–26.00)	0.971	0.038
AST, U/L	31.40 (19.95–69.50)	23.00 (16.00–51.10)	0.062	18.00 (16.00–21.50)	18.00 (15.00–21.98)	17.70 (15.25–21.75)	0.750	0.000
Urea nitrogen, mmol/L	5.36 (4.20–7.18)	5.17 (4.30–6.85)	0.600	5.00 (4.00–6.00)	5.39 (5.00–6.75)	5.39 (5.00–7.00)	0.696	0.963
Serum creatinine, umol/L	86.10 (76.45–95.90)	80.80 (70.40–97.40)	0.302	75.10 (64.25–87.10)	77.15 (67.20–88.55)	78.25 (65.43–89.60)	0.673	0.029
TC, mmol/L	4.34 (3.79–4.89)	4.39 (3.90–5.07)	0.722	3.97 (3.44–4.65)	3.97 (3.38–4.62)	3.91 (3.32–4.54)	0.459	0.005
TG, mmol/L	1.33 (1.05—-1.91)	1.46 (1.11–1.91)	0.425	1.37 (1.06–2.01)	1.38 (1.05–2.15)	1.32 (0.98–1.96)	0.528	0.418
HDL-c, mmol/L	0.98 (0.86–1.17)	0.94 (0.82–1.10)	0.237	1.07 (0.93–1.22)	1.02 (0.90–1.20)	1.06 (0.88–1.17)	0.832	0.031
LDL-c, mmol/L	2.54 (2.16–3.03)	2.58 (2.22–3.11)	0.502	2.16 (1.77–2.71)	2.16 (1.74–2.65)	2.11 (1.74–2.63)	0.592	0.001

P1, comparisons between AMI group and CAE + AMI group; P2, comparisons among CON group, CHD group, and CAE group; P3, comparisons between CAE + AMI group and CAE group.

AMI, acute myocardial infarction; CAE, coronary artery ectasia; CHD, coronary heart disease; CON, coronary artery without CAE and stenosis; SBP, systolic blood pressure; DBP, diastolic blood pressure; HbA1c, hemoglobin A1c; OMI, old myocardial infarction; BMI, body mass index; ALT, glutamic pyruvic transaminase; AST, glutamic-oxaloacetic transaminase; TC, total cholesterol; TG, triglyceride; HDL-c, high-density lipoprotein cholesterol; LDL-c, low-density lipoprotein cholesterol.

*Participants with history of old myocardial infarction were not enrolled in groups without AMI (CON group, CHD group, and CAE group), because our preliminary analysis showed that CAE + AMI was different from CAE (the formal data in this manuscript confirmed it).

As shown in Table 2, the electrolyte, inflammation, cardiac function, and medications were compared among all participants. (1) Comparing the CAE + AMI and AMI groups, hypersensitive C-reactive protein level was higher in the CAE + AMI group than in the AMI group. No further significant differences were observed. (2) The diastolic function indicated by E peak value to A peak (E/A) value was lower in the CAE group than the CHD and CON groups. (3) The CAE + AMI group had a lower left ventricular ejection fraction (LVEF), neutrophil-to-lymphocyte ratio (N/L ratio), and hypersensitive C-reactive protein level than the CAE group.

**TABLE 2:** Electrolyte, inflammation, cardiac function, and medications for all participants.

	AMI (*n* = 153)	CAE + AMI (*n* = 51)	*P1*	CON (*n* = 329)	CHD (*n* = 324)	CAE (*n* = 108)	*P2*	*P3*
N/L ratio	3.42 (2.33–5.42)	2.89 (2.42–4.33)	0.252	2.32 (1.81–3.10)	2.34 (1.83–3.08)	2.36 (1.74–3.02)	0.851	0.000
Neutrophil, 10^9^/L	5.78 (4.26–7.98)	4.99 (3.97–6.52)	0.035	4.12 (3.31–5.04)	4.03 (3.27–4.87)	4.13 (3.18–4.88)	0.932	0.000
Lymphocyte, 10^9^/L	1.66 (1.16–2.27)	1.64 (1.19–2.05)	0.531	1.75 (1.41–2.16)	1.67 (1.36–2.11)	1.71 (1.37–2.14)	0.460	0.148
Hypersensitive C-reactive protein	6.10 (2.29–19.90)	11.00 (7.00–20.00)	0.004	1.41 (0.58–3.46)	1.34 (0.67–3.17)	1.47 (0.65–4.96)	0.491	0.000
Killip ≥ grade II, n (%)	42 (27.45%)	13 (25.49%)	0.785	–	–	–	–	-
peak value of NT-proBNP, pg/ml	1227.00 (430.50–3376.00)	2454.00 (663.50–4711.25)	0.350	–	–	–	–	–
peak value of CK-MB, U/L	27.40 (7.34–78.90)	51.10 (12.85–297.25)	0.293	–	–	–	–	–
peak value of Myo, ug/L	49.90 (31.55–135.50)	116.40 (28.65–455.00)	0.404	–	–	–	–	–
peak value of TnI, ng/ml	3.54 (0.73–10.05)	8.38 (0.56–40.53)	0.275	–	–	–	–	–
LVEF,%	62.00 (55.00–67.75)	62.00 (56.25–67.75)	0.483	68.00 (65.00–71.00)	68.00 (64.00–71.00)	68.00 (65.00–71.00)	0.960	0.000
E/A	0.83 (0.69–1.26)	0.76 (0.67–1.19)	0.349	0.78 (0.67–0.96)	0.79 (0.68–0.94)	0.73 (0.65–0.85)	0.042	0.033
β-blocker, n (%)	97 (63.40%)	34 (66.67%)	0.673	197 (59.88%)	213 (65.74%)	71 (65.74%)	0.252	0.908
ACEI/ARB, n (%)	98 (64.05%)	31 (60.78%)	0.679	149 (45.29%)	166 (51.23%)	54 (50.00%)	0.297	0.203
CCB, n (%)	70 (45.75%)	20 (39.22%)	0.416	133 (40.43%)	137 (42.28%)	47 (43.52%)	0.814	0.608
Statins, n (%)	106 (69.28%)	34 (66.67%)	0.727	295 (89.66%)	291 (89.81%)	99 (91.66%)	0.195	0.000
Uretic, n (%)	19 (12.42%)	4 (7.84%)	0.371	22 (6.69%)	21 (6.48%)	14 (12.96%)	0.066	0.342
Antiplatelet, n (%)	151 (98.69%)	50 (98.04%	0.945	329 (100.00%)	324 (100.00%)	108 (100.00%)	–	0.000
Single antiplatelet, n (%)	6 (3.92%)	2 (3.92%)		–	–	–	–	–
Double antiplatelet, n (%)	145 (94.77%)	48 (94.12%)		–	–	–	–	–

P1, comparisons between AMI group and CAE + AMI group; P2, comparisons among CON group, CHD group, and CAE group; P3, comparisons between CAE + AMI group and CAE group.

AMI, acute myocardial infarction; CAE, coronary artery ectasia; CHD, coronary heart disease; CON, coronary artery without CAE and stenosis; LVEF, Left ventricular ejection fraction; E/A, E peak value to A peak value; N/L ratio, neutrophil-to-lymphocyte ratio; ACEI/ARB, angiotensin-converting enzyme inhibitors and angiotensin receptor blockers; CCB, calcium channel antagonists; CK-MB, creatine kinase-MB fraction; Myo, Myoglobin, NT-proBNP, N-terminal pro-brain natriuretic peptide, TnI, troponin I.

Table 3 shows evaluations of ectasia and stenosis in the coronary arteries. The extent of stenosis, indicated by the Gensini score, and stenosis rate of LCX in the CAE + AMI group were lower than those in the AMI group. For distribution of ectasia, the RCA had the highest involvement with dilatation, and the most common type was Markis type I. Furthermore, the extent of stenosis in the CAE group was similar to that in the CHD group, whereas the stenosis rate of LAD was lower in the CAE group. For the ectasia distribution, RCA had the highest involvement with dilatation, and the most common type was Markis type I. In comparing the CAE + AMI and CAE groups, the CAE + AMI group had a higher stent implant rate, Gensini score, and RCA stenosis and occlusion rates than the CAE group.

**TABLE 3:** Coronary evaluations for ectasia and stenosis.

	AMI (*n* = 153)	CAE + AMI (*n* = 51)	*P*1	CON (*n* = 329)	CHD (*n* = 324)	CAE (*n* = 108)	*P2*	*P3*
Stent, n (%)	122 (79.74%)	39 (76.47%)	0.620	–	175 (54.01%)	55 (50.93%)	0.578	0.000
Gensini score	80.50 (61.00–116.25)	66.00 (43.50–92.00)	0.001	–	46.00 (39.00–56.00)	44.00 (36.00–56.38)	0.450	0.002
LM stenosis, n (%)	19 (12.42%)	7 (13.73%)	0.808	–	31 (9.57%)	12 (11.11%)	0.643	0.635
LAD stenosis, n (%)	143 (93.46%)	47 (92.16%)	0.749	–	320 (98.77%)	99 (91.67%)	0.000	0.916
LCX stenosis, n (%)	112 (73.20%)	45 (88.24%)	0.027	–	240 (74.07%)	78 (72.22%)	0.705	0.024
RCA stenosis, n (%)	153 (100.00%)	50 (98.04%)	0.083	–	248 (76.54%)	87 (80.56%)	0.387	0.003
LM occlusion, n (%)	(0.00%)	(0.00%)	–	–	0 (0.00%)	0 (0.00%)	–	–
LAD occlusion, n (%)	23 (15.03%)	4 (7.84%)	0.189	–	10 (3.09%)	3 (2.78%)	0.877	0.146
LCX occlusion, n (%)	21 (13.73%)	6 (11.76%)	0.720	–	15 (4.63%)	7 (6.48%)	0.448	0.256
RCA occlusion, n (%)	66 (43.14%)	16 (31.37%)	0.138	–	13 (4.01%)	6 (5.56%)	0.498	0.000
LM ectasia, n (%)	–	3 (5.66%)	–	–	–	15 (13.89%)	–	0.137
LAD ectasia, n (%)	–	14 (26.42%)	–	–	–	23 (21.30%)	–	0.391
LCX ectasia, n (%)	–	17 (32.08%)	–	–	–	36 (33.33%)	–	1.000
RCA ectasia, n (%)	–	34 (64.15%)	–	–	–	70 (64.81%)	–	0.819
Markis classification								0.037
Type I	–	24 (45.28%)	–	–	–	41 (37.96%)	–	
Type II	–	11 (20.75%)	–	–	–	11 (10.19%)	–	
Type III	–	13 (24.53%)	–	–	–	36 (33.33%)	–	
Type IV	–	3 (5.66%)	–	–	–	20 (18.52%)	–	

P1, comparisons between AMI group and CAE + AMI group; P2, comparisons among CON group, CHD group, and CAE group; P3, comparisons between CAE + AMI group and CAE group.

AMI, acute myocardial infarction; CAE, coronary artery ectasia; CHD, coronary heart disease; CON, coronary artery without CAE and stenosis; LM, left main coronary artery; LAD, left anterior descending coronary artery; LCX, left circumflex coronary artery; RCA, right coronary artery.

**FIGURE 2:**
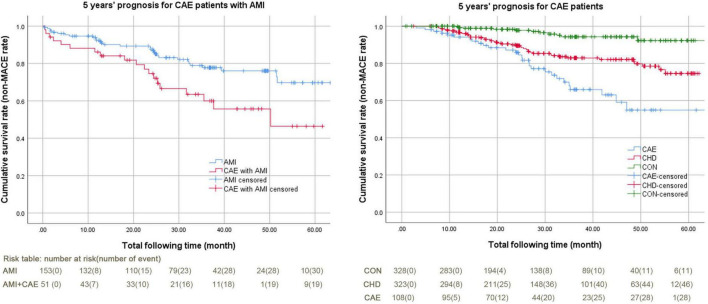
Kaplan-Meier curves of 5-year MACE for patients with CAE + AMI and CAE. AMI, acute myocardial infarction; CAE, coronary artery ectasia; CHD, coronary heart disease; CON, coronary artery without CAE and stenosis; MACE, major adverse cardiovascular events. The MACE in this study included cerebral infarction, cerebral hemorrhage, cardiac death, major hemorrhagic events, malignant arrhythmias, cardiogenic shock, readmission, revascularization therapy, and AMI. 1) **Figure left:** Kaplan-Meier curves of 5-year MACE for patients with CAE + AMI, log Rank (Mantel-Cox) *P* value was 0.010. The non-MACE percentages were 79.70% and 62.70%; the non-MACE time spans were 53.37 (49.66–57.08) months and 41.91 (35.14–48.67) months. The total follow-up times were 32.07 (13.20–48.87) and 25.33 (13.37–42.80) months. 2) **Figure right:** Kaplan-Meier curves of 5-year MACE for patients with CAE, Log Rank (Mantel-Cox) *P* value was 0.000. The non-MACE percentages were 96.70%, 85.80%, and 74.10%. The non-MACE time spans were 61.84 (60.38–63.29) months, 54.08 (51.96–56.20) months, and 49.75 (44.58–54.92) months. The total follow-up times were 24.10 (12.53–42.85) months, 27.50 (15.50–46.77) months, and 26.62 (15.63–36.57) months.

Figure 2 shows the 5-year prognostic analysis of all participants using the Kaplan-Meier curve. The overall prognosis of the CAE + AMI group was worse than that of the AMI group (cumulative survival rate or 5-year non-MACE rate: 62.70% vs. 79.70%, *P* = 0.010) (Figure 1, left). The overall prognosis of the CAE group was worse than those of the CHD and CON groups (cumulative survival rate or 5-year non-MACE rate: 74.10% vs. 85.80% and 96.70%, respectively, *P* = 0.000) (Figure 1, right).

Regarding MACE in the CAE + AMI and AMI groups, the main MACE in the CAE + AMI group was AMI reoccurrence (19.61% vs. 4.57%, *P* = 0.002). Comparing MACE in the CAE, CHD, and CON groups, the main MACE in the CAE group was re-hospitalization due to repeated angina pectoris (14.81% vs. 8.33% and 2.74%, *P* = 0.000). The MACE rates in the CAE + AMI and CAE groups were similar, but the exact MACE events differed between the groups [AMI including STEMI and NSTEMI (3.70% vs. 19.61%) and re-hospitalization due to repeated angina pectoris (14.81% vs. 9.80%)] (Table 4).

**TABLE 4:** The MACE in CAE + AMI population and in CAE population.

	AMI (*n* = 153)	CAE + AMI (*n* = 51)	*P1*	CON (*n* = 329)	CHD (*n* = 324)	CAE (*n* = 108)	*P2*	*P3*
Tatal MACE, n (%)	31 (20.26%)	19 (37.25%)	0.015	11 (3.34%)	46 (14.20%)	28 (25.93%)	0.000	0.144
Cerebral infarction, n (%)	2 (1.31%)	0 (0.00%)	0.002	0 (0.00%)	2 (0.62%)	0 (0.00%)	0.000	0.013
Cerebral hemorrhage, n (%)	1 (0.65%)	0 (0.00%)		0 (0.00%)	0 (0.00%)	0 (0.00%)		
Death, n (%)	7 (4.58%)	3 (5.88%)		0 (0.00%)	3 (0.93%)	3 (2.78%)		
Gastrointestinal bleeding, n (%)	1 (0.65%)	0 (0.00%)		0 (0.00%)	0 (0.00%)	1 (0.93%)		
Malignant arrhythmia, n (%)	0 (0.00%)	0 (0.00%)		0 (0.00%)	1 (0.31%)	0 (0.00%)		
Cardiogenic shock, n (%)	0 (0.00%)	1 (1.96%)		0 (0.00%)	0 (0.00%)	0 (0.00%)		
Rehospitalization due to UAP, n (%)	12 (7.84%)	5 (9.80%)		9 (2.74%)	27 (8.33%)	16 (14.81%)		
Revascularization, n (%)	1 (0.65%)	0 (0.00%)		0 (0.00%)	10 (3.09%)	3 (2.78%)		
STEMI, n (%)	6 (3.92%)	2 (3.92%)		0 (0.00%)	0 (0.00%)	2 (1.85%)		
NSTEMI, n (%)	1 (0.65%)	8 (15.69%)		2 (0.60%)	3 (0.93%)	2 (1.85%)		

P1, comparisons between AMI group and CAE + AMI group; P2, comparisons among CON group, CHD group, and CAE group; P3, comparisons between CAE + AMI group and CAE group.

AMI, acute myocardial infarction; CAE, coronary artery ectasia; CHD, coronary heart disease; CON, coronary artery without CAE and stenosis; MACE, major adverse cardiovascular events, including cerebral infarction, cerebral hemorrhage, cardiac death, major hemorrhagic events, malignant arrhythmias, cardiogenic shock, readmission, revascularization therapy, AMI; STEMI, ST-segmental elevated myocardial infarction; non-STEMI, non-ST-segmental elevated myocardial infarction.

After the COX regression analyses of the CAE + AMI and AMI groups, the group (AMI = 0, CAE + AMI = 1) was found to be a promoting factor for MACE. For the CAE, CHD, and CON groups, the group (control = 1, CHD = 2, CAE = 3) was found to be a promoting factor for MACE (Table 5).

**TABLE 5:** Cox regression for MACE (all participants).

		Coefficient Beta	Standardization error	Wald	*P*-value	Standardization coefficient Beta with 95% CI
AMI, CAE + AMI	Group (AMI = 0, CAE + AMI = 1)	0.784	0.296	6.989	0.008	2.189 (1.225–3.914)
	ACEI/ARB, n (%)	–0.603	0.293	4.241	0.039	0.547 (0.308–0.971)
	Statins, n (%)	–0.597	0.294	4.110	0.043	0.551 (0.309–0.98)
	Killip grade, n (%) (I = 0, ≥ II = 1)	0.004	0.001	11.915	0.001	1.004 (1.002–1.007)
CON, CHD, CAE	Gensini score	0.009	0.003	7.301	0.007	1.009 (1.003–1.016)
	LAD stenosis, n (%)	1.022	0.438	5.453	0.020	2.779 (1.178–6.551)
	group (CON = 1, CHD = 2, CAE = 3)	0.631	0.198	10.166	0.001	1.879 (1.275–2.768)

AMI, acute myocardial infarction; CAE, coronary artery ectasia; CHD, coronary heart disease; CON, coronary artery without CAE and stenosis; CI, confidence interval; ACEI/ARB, angiotensin-converting enzyme inhibitors and angiotensin receptor blockers; LAD, left anterior descending coronary artery.

The upper part was analyzed in the AMI population (AMI group and CAE + AMI group); the lower part was analyzed in the non-AMI population (CON group, CHD group, and CAE group). In this setting, MACE was the dependent variable, and other factors that may relate to MACE were arranged as independent variables. They included age, years, sex, hypertension, diabetes, smoking, alcohol, BMI, hepatic and renal functions, lipid profiles, lactic acid, carbon dioxide, anion gap, osmotic pressure, N/L ratio, hypersensitive C-reactive protein, medications, stent implant, Gensini score, disruption of stenosis, and occlusion and ectasia in coronary arteries.

Based on the COX regression analyses, 1) the protective factors preventing MACE in the CAE + AMI group were antiplatelet agents (standardization coefficient beta or hazard ratio = 0.234, *P* = 0.016) and ACEI/ARB (standardization coefficient beta or hazard ratio = 0.317, *P* = 0.037). 2) For the CAE group, the main factor promoting MACE was the degree of coronary stenosis (Gensini score, hazard ratio = 1.011, *P* = 0.022) (Table 6).

**TABLE 6:** Cox regression for MACE (patients with CAE).

		Coefficient Beta	Standardization error	Wald	*P*-value	Standardization coefficient Beta with 95% CI
In CAE + AMI group	ACEI/ARB, n (%)	–1.415	0.589	5.773	0.016	0.243 (0.077–0.771)
	TG, mmol/L	–2.008	0.702	8.170	0.004	0.134 (0.034–0.532)
	Killip grade[I = 0, ≥ II = 1 n (%)]	0.011	0.003	11.993	0.001	1.011 (1.005–1.017)
	Serum creatinine, umol/L	0.012	0.007	3.449	0.063	1.012 (0.999–1.026)
	Antiplatelet, n (%)	–1.148	0.550	4.357	0.037	0.317 (0.108–0.932)
In CAE group	Gensini score	0.010	0.004	4.528	0.033	1.010 (1.001–1.018)

AMI, acute myocardial infarction; CAE, coronary artery ectasia; CHD, coronary heart disease; CON, coronary artery without CAE and stenosis; CI, confidence interval; ACEI/ARB, angiotensin-converting enzyme inhibitors and angiotensin receptor blockers; TG, triglyceride.

The upper part was analyzed in the CAE + AMI group; the lower part was analyzed in the CAE group. In this setting, MACE was the dependent variable, and other factors that may relate to MACE were arranged as independent variables. They included age, years, sex, hypertension, diabetes, smoking, alcohol, BMI, hepatic and renal functions, lipid profiles, lactic acid, carbon dioxide, anion gap, osmotic pressure, N/L ratio, hypersensitive C-reactive protein, medications, stent implant, Gensini score, disruption of stenosis, and occlusion and ectasia in coronary arteries.

The purpose of logistic regression was aimed to find out the independent factors relating to CAE + AMI (CAE = 0, CAE + AMI = 1). It revealed that N/L ratio, LDL-c, and hypersensitive C-reactive protein level were positively associated with the CAE + AMI, whereas Markis type was negatively associated with the CAE + AMI (Table 7).

**TABLE 7:** Logistic regression analysis for patients with CAE and CAE + AMI.

	Coefficient Beta	Standardization error	Wald	*P*-value	Standardization coefficient Beta with 95% CI
N/L ratio	0.533	0.214	6.174	0.013	1.703 (1.119–2.593)
LDL-c, mmol/L	0.930	0.305	9.308	0.002	2.535 (1.395–4.608)
Hypersensitive C-reactive protein	0.163	0.044	13.455	0.000	1.177 (1.079–1.284)
Markis classification (I, II, III, IV)	–0.593	0.249	5.673	0.017	0.553 (0.339–0.900)
Constant	–4.479	1.073	17.435	0.000	

AMI, acute myocardial infarction; CAE, coronary artery ectasia; LDL-c, low-density lipoprotein cholesterol; N/L ratio, neutrophil-to-lymphocyte ratio; CI, confidence interval.

In this setting, CAE + AMI and CAE were the dependent variables, and other factors that may relate to AMI were arranged as independent variables. They included age, years, sex, hypertension, diabetes, smoking, alcohol, BMI, hepatic and renal functions, lipid profiles, lactic acid, carbon dioxide, anion gap, osmotic pressure, N/L ratio, hypersensitive C-reactive protein, Gensini score, disruption of stenosis, and occlusion and ectasia in coronary arteries. The medications were not previously presented in this study. Therefore, they were not included in this logistic regression model. The items that might occur after AMI were also included in this model.

## Discussion

It was lack of the overall prognosis for patients with CAE and those with CAE + AMI (15). Therefore, this study enrolled a consecutive series of 51 patients with CAE + AMI and 108 patients with CAE + CHD. They were matched with controls at a 1:3 ratio using the propensity score method and were followed up. From the study findings, the Kaplan-Meier curve showed that the MACE of participants in the CAE + AMI group was higher than those in the AMI group. Furthermore, the MACE of participants in the CAE group was higher than that of those in the CHD and CON groups. Regarding MACE, the main MACEs in the CAE + AMI and CAE groups were AMI reoccurrence and re-hospitalization due to repeated angina pectoris, respectively. Additionally, the COX regression analysis revealed that the protective factors for preventing MACE in the CAE + AMI group included antiplatelet agents and ACEI/ARB while the main factor promoting MACE in the CAE group was the degree of coronary stenosis. The logistic regression analysis showed that the factors with positive association with CAE + AMI were higher inflammatory status and poorer lipid profile, whereas that with negative association with CAE + AMI was Markis types.

First, the overall prognosis of patients with CAE was poorer than that of patients with CHD. Additionally, the prognosis of patients with CAE + AMI was poorer than patients with AMI. The relationship between CAE and CHD remained unclear, although more than 80% of patients with CAE had comorbid CHD (16). According to some studies, CAE is a variation of CHD because the two diseases have similar features, including risk factors, clinical symptoms, and pathological findings (16, 17). Both diseases have obvious atherosclerotic changes in the intima and media of the coronary artery. However, the damage of the middle coronary artery is an important difference between CHD and CAE (4, 5). An obvious slow coronary blood flow and microcirculation are present in dilated coronary arteries (6). Therefore, the overall prognosis of patients with CAE was understandably poorer than that of patients with CHD, because those extra characters might contribute to the extra MACE in patients with CAE. The prognostic data were useful for designing the treatment strategy, although no consensus on the treatment for patients with CAE was not reached. Currently, most of the treatment proposals for CAE are based on CHD treatment (18, 19). Therefore, to decrease MACE in patients with CAE, this study indicated the need for more aggressive treatments for patients with CAE than for those with CHD.

Second, the CAE and CAE + AMI groups were found to be different populations. Patients with CAE had a higher risk of repeated angina pectoris than patients with AMI. Patients with CAE + AMI had a higher risk of AMI reoccurrence but not angina pectoris (20–22). The underlying mechanism contributing to these differences was unclear. The logistic regression in this study revealed that the factors positively associated with AMI in the CAE population were higher inflammatory status and poor lipid profiles. As known, CAE is an inflammatory disease (6, 23); therefore, a higher inflammatory status might be predispose patients to occurrence of thrombotic events. Other underlying factors relating to AMI in patients with CAE should be explore further. In this study, for patients with CAE, more aggressive treatment was needed to relieve ischemia and ischemic symptoms but not to prevent thrombosis. Whereas for patients with CAE + AMI, more aggressive treatment was needed to prevent thrombosis but not to relieve ischemia and ischemic symptoms. These findings are helpful in categorizing patients with CAE and formulating specific treatment strategies in future clinical practice, because for a long period, CAE was not categorized into different subgroups according to their different prognoses.

Third, the detailed treatment strategy for patients with CAE was explored in this study. From the perspective of MACE prevention, the progression of atherosclerotic lesions should be prevented in patients with CAE (23, 24) while the anti-thrombotic treatments, are indicated for patients with CAE + AMI (25, 26). As reported in our previous study, patients with CAE and CHD had minimal ectasia progression but the atherosclerosis progressed gradually (3). The dynamic change of CAE might be mainly manifested by the development of atherosclerotic changes but not the extent of ectasia; therefore, for patients with CAE, the main treatment might be to prevent the progression of atherosclerotic lesions; for CAE + AMI patients, the thrombotic events might be related to ectasia itself (higher inflammatory status and poor lipid profiles) or other unknown factors (23), and the remodeling of left ventricular could be improved by ACEI/ARBs. Most medicines for CAE are based on the current treatment options for CHD, such as antithrombotic therapy, statins, and trimetazidine (10). According to our findings, anti-atherosclerotic agents, including statins, should be more aggressively used in patients with CAE. For patients with CAE + AMI, anti-thrombotic treatment options, including double anti-platelet agents, or a more aggressive strategy by combination of anti-platelet agents and anti-coagulation agents should be considered (27, 28). Additionally for patients with poor response to medications, coronary artery interventional therapy and coronary artery bypass graft should be considered (1).

This study had the following limitations. First, the small sample size was due to the difficulty in recruiting participants, owing to the low prevalence of CAE and use of special diagnostic methods, such as coronary angiography, but not other routine methods. Second, this study was a single-center study. Therefore, further multi-center collaborations are needed in the future to produce more representative results.

## Conclusion

The prognosis of patients with CAE + AMI were worse than that of patients with AMI, and the overall prognosis for patients with CAE patients was worse than that of patients with CHD. The CAE + AMI and CAE groups had different characteristics; the former was prone to AMI reoccurrence, and the latter was prone to repeated angina pectoris. To prevent MACE, medications, including antiplatelets and ACEI/ARBs, are indicated for patients with CAE + AMI, whereas prevention of the progression of atherosclerotic lesions is indicated for patients with CAE.

## Data availability statement

The original contributions presented in this study are included in the article/supplementary material, further inquiries can be directed to the corresponding author.

## Ethics statement

The studies involving human participants were reviewed and approved by the Ethics committee of Beijing Friendship Hospital Affiliated to Capital Medical University. The patients/participants provided their written informed consent to participate in this study.

## Author contributions

RL and HZ: conceive the study and its design, conceptualization, formal analysis, resources, and software. HZ: data curation. RL, XG, SL, and HZ: investigation and validation. XG: methodology. RL, SL, and HZ: project administration. All authors contributed to the article and approved the submitted version.
